# The Basophilic Fringe: A Case Report of Intestinal Spirochetosis in a Patient With Chronic Diarrhea

**DOI:** 10.7759/cureus.102724

**Published:** 2026-01-31

**Authors:** David Horvath, Laurentia Nodit

**Affiliations:** 1 Department of Pathology, The University of Tennessee Health Science Center College of Medicine, Knoxville, USA

**Keywords:** chronic diarrhea, histopathology, intestinal spirochetosis, largely unremarkable colonoscopy, special and immunohistochemistry staining

## Abstract

Intestinal spirochetosis is a rare infection of the colonic mucosa that often presents with nonspecific gastrointestinal symptoms despite largely normal endoscopic findings. Diagnosis depends on characteristic histologic features supported by immunohistochemical staining. Here, we present the case of a 57-year-old man with HIV, chronic abdominal pain, bloating, and long-standing watery diarrhea. His colonoscopy was largely unremarkable except for rectal ulcers. Histological examination of random colonic biopsies revealed a basophilic, brush-like fringe along the epithelial surface consistent with intestinal spirochetosis, which demonstrated immunoreactivity with anti-*Treponema pallidum* staining. Treatment with metronidazole led to the complete resolution of symptoms. This case underscores the importance of considering intestinal spirochetosis in patients with chronic diarrhea and abdominal pain when endoscopic findings are nonspecific, as prompt histologic diagnosis allows for effective treatment.

## Introduction

Intestinal spirochetosis is characterized by infection of the colonic mucosa with spirochete species and is infrequently identified, with an overall prevalence of approximately 1% in colonic biopsies, increasing in regions with lower standards of living [1,2]. Although not entirely clear, the pathophysiology is believed to be related to mucosal disruption by adherent spirochetes [1,2].

Two species, *Brachyspira aalborgi and Brachyspira pilosicoli*, are acknowledged as the primary causative organisms in intestinal spirochetosis. Patients with *B. pilosicoli* are more likely to have diarrhea, be HIV positive or immunocompromised, and experience resolution of symptoms following treatment with metronidazole than patients with *B. aalborgi* infection [3]. While infection by either species is often asymptomatic, the clinical picture can include vague abdominal pain, watery diarrhea, hematochezia, and bloating [4]. The endoscopic appearance of the colon is widely nonspecific, with findings that may include erosions, polypoid lesions, or erythematous mucosa [4]. Histologically, this infection manifests itself as a basophilic brush-like border that edges the colonic epithelium. Diagnosis is based on the identification of this basophilic fringe and supplemented by immunohistochemical stains, such as Warthin-Starry, that highlight the spirochete colonization. This case emphasizes the critical role of histological review in HIV-positive patients with chronic diarrhea and nonspecific endoscopic findings.

## Case presentation

A 57-year-old man presented to a gastroenterologist with a complaint of chronic bilateral lower quadrant, dull abdominal pain associated with bloating. Additionally, these symptoms have coincided with years of watery diarrhea that the patient reports as types 5, 6, and 7 on the Bristol stool scale [5]. Medical history is unremarkable except for HIV with a CD4 count of 482 cells/µL, chronic abdominal pain, bloating, and long-standing watery diarrhea. The patient underwent further evaluation with an upper and lower endoscopy. Upper endoscopy revealed an unremarkable esophageal and gastric mucosa. The colonic mucosa was normal except for two morphologically similar, nonspecific rectal ulcers measuring 15 mm in greatest dimension. Due to concern for a syphilitic ulcer, a rapid plasma reagin test was ordered and returned negative.

**Figure 1:**
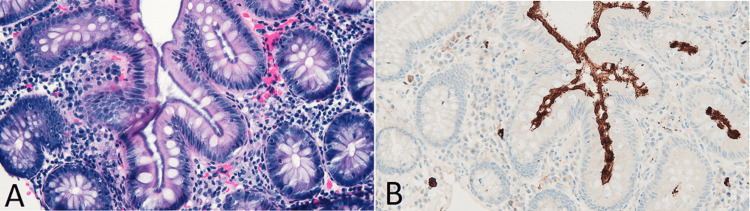
(A) H&E stain showing a basophilic fuzz on the luminal surface of epithelial cells. (B) An anti-Treponema pallidum stain gives the spirochetes a brown tincture, allowing one to visualize these bacteria on the epithelial surface and between adjacent crypts H&E: hematoxylin and eosin

Histological evaluation of a random cecal and ascending colon biopsy revealed a 2-3-µm-thick basophilic band of filamentous spirochetes (Figure 1A). A mild increase in plasma cells is apparent within the lamina propria. An anti-Treponema pallidum immunohistochemical stain was used to accentuate the spirochetes on the colonic surface (Figure 1B). The rectal biopsies did not show histological or immunohistochemical evidence of spirochetes.

Based on the clinical history and diagnostic findings, the patient was prescribed metronidazole, 500 mg orally, three times daily for 10 days. Initial symptom relief was noted by day 7 with complete resolution by day 10. Given this response, an interval colonoscopy was scheduled in 10 years.

## Discussion

Intestinal spirochetosis is a rare infection of the colonic mucosa commonly caused by *Borrelia eurygyrata, B. aalborgi, or Serpulina pilosicoli *[1]. While often asymptomatic, it can present with vague abdominal pain, bloating, and diarrhea, as seen in this 57-year-old man with a history of HIV [4]. Like the observations in this case, the endoscopic findings are largely nonspecific. However, some abnormalities, such as an inflamed hyperemic mucosa and stigmata of recent bleeding, have been reported. Histologically, the infection is characterized by a hyperchromatic fuzz of spirochetes adhering to the epithelium. The use of stains, such as anti-*Treponema pallidum*, Giemsa, periodic acid-Schiff, and silver stains such as Warthin-Starry or Grocott, can enhance the identification of the spirochetes and confirm the diagnosis [4,6].

The pathogenic mechanisms of Brachyspira are not entirely elucidated, but the bacteria are believed to adhere to and disrupt the mucosal barrier, which may contribute to the clinical symptoms, including diarrhea and abdominal discomfort [1].

The infection is associated with states of immunosuppression, inflammatory bowel disease, men who have sex with men, and malnutrition [3]. Although HIV was well controlled in this patient, intestinal spirochetosis is usually observed in HIV-infected individuals with CD4 cell counts above 200/mL. The infection can also occur in asymptomatic, otherwise healthy children.

Treatment of intestinal spirochetosis generally involves antibiotics, with metronidazole being the most commonly used agent [3]. This infection responds well to metronidazole, making the prognosis excellent. Tetracyclines and erythromycin are other options, though metronidazole remains the first-line choice in most clinical scenarios. While the clinical outcome was not available, this antibiotic regimen is generally effective in resolving symptoms. Follow-up to assess resolution of symptoms is important, as immunocompromised patients may need repeated courses of treatment.

## Conclusions

Intestinal spirochetosis should be considered in patients with chronic diarrhea and abdominal pain with nonspecific endoscopic findings. Diagnosis relies on histological and immunohistochemical identification of spirochetes. Treatment with metronidazole typically leads to symptom resolution. Given the rarity of the condition, we hope this case will raise awareness of this infectious entity, leading to a timelier diagnosis and treatment.
